# Phenolic Profile and Fingerprint Analysis of *Akebia quinata* Leaves Extract with Endothelial Protective Activity

**DOI:** 10.3390/molecules27144636

**Published:** 2022-07-20

**Authors:** Dan Gao, Chong-Woon Cho, Jin-Hyeok Kim, Haiying Bao, Hyung-Min Kim, Xiwen Li, Jong-Seong Kang

**Affiliations:** 1 Institute of Chinese Materia Medica, China Academy of Chinese Medical Sciences, Beijing 100700, China; gaodan521361@hotmail.com; 2 College of Pharmacy, Chungnam National University, Daejeon 34134, Korea; chongw113@naver.com (C.-W.C.); oojh52@cnu.ac.kr (J.-H.K.); kimhm@cnu.ac.kr (H.-M.K.); 3 College of Chinese Medicinal Materials, Jilin Agriculture University, Changchun 130118, China; baohaiying2008@126.com

**Keywords:** endothelial dysfunction, *Akebia quinata* leaves, phenolic compound, human umbilical vein endothelial cell, fingerprint, quality control

## Abstract

In contrast to the stem and fruit of *Akebia quinata*, *A. quinata* leaves as a source rich in phenolic compounds with potentially beneficial pharmacological activities have been largely overlooked. To develop and use *A. quinata* leaves as a resource, we evaluated its potential as a cardiovascular-protective agent. Herein, we investigated the effects and potential mechanisms of *A. quinata* leaves extract on lipopolysaccharide (LPS)-induced inflammatory responses in human umbilical vein endothelial cells. We found that *A. quinata* leaves extract pretreatment of 10 μg/mL significantly attenuated LPS-induced protein expression of intercellular adhesion molecule-1, vascular cell adhesion molecule-1. Furthermore, this extract also suppressed LPS-induced phosphorylation of nuclear factor-κB p65. In order to elucidate the chemical profiles of the samples, the HPLC fingerprint was established, and prominent peaks were identified via HPLC–electrospray ionization–mass spectrometry. Multivariate statistical analyses, including hierarchical cluster analysis, principal component analysis, and partial least-squares discriminant analysis, were performed to evaluate the clustering of the samples. It was found that isochlorogenic acid C was a key marker for the classification of *A. quinata* leaves from the Gongju and Muju city in Korea. Collectively, this study not only suggested the potential of *A. quinata* leaves as a novel therapeutic candidate for inflammatory cardiovascular disease but also developed a quality control method for *A. quinata* leaves, which could help to expand the application of *A. quinata*.

## 1. Introduction

As the major surface membrane component in many Gram-negative bacteria, lipopolysaccharide (LPS) could induce dysfunction of endothelial cells and play a central role in the initiation of the inflammatory process in miscellaneous cardiovascular diseases [1,2,3]. The endothelial inflammatory response increases vascular permeability and promotes leukocyte adhesion by increasing the overproduction of several cell adhesion molecules, including intercellular adhesion molecule-1 (ICAM-1), vascular cell adhesion molecule 1 (VCAM-1), and E-selectin [4]. Meanwhile, the activation of nuclear factor-κB (NF-κB) is known as a crucial step in the inflammatory response, as it regulates the expression of various inflammatory mediators that upturn endothelial permeability and uphold cell adhesion [5,6]. Therefore, evaluating the effects of potential herb extracts or compounds on the downregulation of the expression of adhesion molecules as well as inflammatory cytokines, and inhibiting the NF-κB pathway, could be used to screen promising candidates used in the treatment and prevention of atherosclerotic diseases.

*Akebia quinata* (Houtt.) Decne., known as five-leaf Akebia, is widely distributed in Korea, China, and Japan [7,8]. Dried *A. quinata* stems are traditionally used as a diuretic agent for the treatment of hypothermia and rheumatic pain, as well as a sedative, while the fruits of *A. quinata* are generally used as antineoplastic, antiphlogistic, and antidiuretic agents in traditional Chinese medicines [9,10,11,12,13]. Currently, most of the research on *A. quinata* has focused on the biological and phytochemical characteristics of the stems and fruits rather than the leaves [14,15,16]. Interestingly, *A. quinata* leaves are registered as a food in the Korean food safety database, indicating that the leaves could be used as a raw material for developing functional foods [17,18]. Among the public, *A. quinata* leaves are blanched and used as a food to treat obesity and cardiovascular disease [19]. In a recent study, young leaves of *A. quinata* have been proved that exerts a beneficial anti-obesity effect in mice by decreasing the levels of intracellular triglycerides as well as free glycerol and suppressing lipid accumulation in 3T3-L1 fat cells [19]. However, the potential mechanism of *A. quinata* leaves extracts to avoid LPS-induced endothelial inflammatory responses is poorly investigated.

The simultaneous determination of multiple bioactive components has been a recent developing trend in evaluating the quality of natural products; however, no effective HPLC method has been reported to comprehensively evaluate the quality of *A. quinata* leaves. Some published analytical methods that only control the quality of the fruits and stems of *A. quinata* include HPLC-ultraviolet (HPLC–UV) detection method and HPLC coupled with electrospray ionization-quadrupole-time-of-flight mass spectrometry (HPLC–ESI–Q-TOF–MS-MS) [20,21,22]. Unfortunately, none of these established methods can be used to identify and quantify the active ingredients in *A. quinata* leaves because the distribution of the individual metabolites varies greatly in the different parts of *A. quinata*.

In the present study, we evaluated the potential role of *A. quinata* leaves extracts in regulating the expression levels of ICAM-1, VCAM-1, and inflammatory processes by LPS-stimulated human umbilical vein endothelial cells (HUVECs), a generally used cell model of vascular inflammation. Meanwhile, we applied an HPLC method with diode-array detection (HPLC–DAD) and HPLC–MS to identify the bioactive components of *A. quinata* leaves by comparing the retention time, UV absorption spectrum, fragment ions, and relative abundance with standards and references. Further, a simple HPLC–DAD method was established for the simultaneous quantitative analysis of the bioactive components found in *A. quinata* leaves for the first time. Additionally, fingerprints combined with multivariate statistical analyses, including heatmap analysis, principal component analysis (PCA), and partial least-squares discriminant analysis (PLS-DA), were applied to identify and evaluate the chemical differences in 14 leaves samples harvested from the Gongju and Muju city in Korea.

## 2. Materials and Methods

### 2.1. Plant Materials and Reagents

A total of 14 *A. quinata* leaves samples were collected from different areas in Gongju and Muju, South Korea. All samples were authenticated by Professor Jong Seong Kang (College of Pharmacy, Chungnam National University, Daejeon, Korea), as shown in Figure S1 and Table S1 (Supplementary Materials). The samples were deposited in the pharmaceutical analysis laboratory, College of Pharmacy, Chungnam National University, with a code number of AQLS. The harvested samples were dried in an LTO-Do-150S oven (Labtech-one, Daejeon, Korea) at 45 °C for 3 days. After drying, the samples were pulverized into a fine powder, then stored at 4 °C until use. Mouse anti-ICAM-1 (#sc-8439), mouse VCAM-1 (#sc-13160), mouse anti-NF-kB p65 (sc-514451), and anti-phospho-eNOS antibody were purchased from Santa Cruz Technology, Inc. (Santa Cruz, CA, USA). The reference standards of neochlorogenic acid, chlorogenic acid, cryptochlorogenic acid, rutin isochlorogenic acid C, 3-(4,5-dimethylthiazol-2-yl)-2,5-diphenyltetrazolium bromide (MTT), and a buffer solution of formic acid were purchased from Sigma-Aldrich Chemical Co. (St. Louis, MO, USA). HPLC-grade methyl alcohol and acetonitrile were purchased from Burdick & Jackson (Muskegon, MI, USA). Water was purified using a Milli-Q system (Milford, MA, USA).

### 2.2. Extraction Optimization

The variables affecting the extraction yields were optimized to achieve efficient extraction of the active compounds. Ultrasound-assisted extraction (UAE) is the preferred extraction method due to its environment-friendly, energy-saving, and time-saving properties. As such, the UAE conditions were optimized by adjusting the extraction time (15, 30, 45, 60, 80, 100, and 120 min) and the sample (g) to solvent (mL) ratio (1:10, 1:20, 1:40, 1:80, and 1:160). The power of the ultrasonic treatment was set at 280 W. The 70% ethanol extract solution was centrifuged for 5 min at 4000 rpm, and then the obtained supernatant was filtered by a 0.22 μm syringe filter before HPLC analysis. In addition, the obtained supernatant was dried and redissolved into different concentrations for subsequent activity evaluation experiments.

### 2.3. Cell Culture

HUVECs were isolated from collagenase-digested umbilical cord veins and collected in M200 medium supplemented with LSGS (Cascade Biologics, Inc., Portland, OR, USA) and 2% fetal bovine serum (FBS, Atlanta Biologicals, Inc., Lawrenceville, GA, USA) [23]. HUVECs were cultured in Petri dishes coated with 0.2% gelatin type A (#901771; MP Biomedicals, Santa Ana, CA, USA) in Endothelial Cell Medium (ECM, #1001, ScienCell, Carlsbad, CA, USA). The media was supplemented with 5% (*v*/*v*) FBS (#0025, ScienCell, Carlsbad, CA, USA), 5 mL of Endothelial Cell Growth Supplement (ECGS, #1052, ScienCell, Carlsbad, CA, USA) and 5 mL of penicillin/streptomycin (P/S, #0503, ScienCell, Carlsbad, CA, USA). All cells were maintained in culture at 37 °C in a 5% CO_2_ humidified atmosphere. 

### 2.4. Cell Viability Assay

The viability of HUVECs was carried out using the MTT assay. Cells were cultured at the density of 1 × 10^4^ cells/well in a 96-well plate. After 24 h of incubation, the cells were starved with the fresh FBS-free medium for 24 h. The adhered cells were treated with different concentrations (0, 1, 2.5, 5, and 10 μg/mL) of the extracts for 24 h. MTT (12 mM) in FBS-free Dulbecco’s modified eagle medium was prepared to treat in each well, and the cells were incubated for 2 h at 37 °C and 5% CO_2_. The medium was removed, and the formazan precipitate was extracted in DMSO and further measured at 540 nm on a microplate reader (TECAN, Männedorf, Switzerland).

### 2.5. Western Blotting

HUVECs were pretreated with vehicle, or *A. quinata* leaves extracts (10 μg/mL) and followed by treatment with 200 ng/mL of LPS. Afterward, cells were washed with PBS, and lysates were prepared using 2× SDS lysis buffer including 1 M Tris-HCl (pH 7.4), 10% SDS, 25% glycerol, 5% 2-mercaptoethanol, and 1% bromphenol blue [24]. The protein extracts were resolved by using sodium dodecyl sulfate-polyacrylamide gel electrophoresis. Following blocking with 5% skimmed milk for 1 h at ambient temperature, the membranes were incubated with primary antibodies (1:1000 dilution) at 4 °C for 12 h, and subsequently incubated with secondary antibodies (1:2500 dilution) for 90 min at ambient temperature. The proteins on the membrane are eventually visualized by using the enhanced chemiluminescence detection reagents (Amersham Pharmacia Biotech, Piscataway, NJ, USA) based on the manufacturer’s instructions. The expression level of each protein was measured by western blotting with each matching unique antibody.

### 2.6. HPLC–DAD–ESI–MS/MS Analysis

The bioactive components in the *A. quinata* leaves were identified using a triple quadrupole mass spectrometer (Shimadzu, Kyoto, Japan) connected to a Prominence TM HPLC system with an ESI source. Chromatographic separation was performed using an RStech HECTOR-M C18 column (250 × 4.6 mm, 5 μm; Daejeon, Korea) with a column temperature of 30 °C. The mobile phase consisted of water (A; containing 0.1% formic acid), acetonitrile (B; containing 0.1% formic acid), and a flow rate of 0.5 mL/min. The gradient condition was optimized as follows: 0–100 min, 10–30% B. The mass spectrometry was carried out in both negative and positive modes with an interface voltage of −3.5 kV and 3.5 kV, respectively.

### 2.7. Quality Control of A. Quinata Leaves

HPLC analysis was performed on a Shimadzu HPLC system equipped with DAD. All tested samples were eluted through an RStech HECTOR-M C18 column (250 × 4.6 mm, 5 μm; Daejeon, Korea) at a flow rate of 1 mL/min. The mobile phase was the same as the LC–MS analysis condition, and the gradient condition was optimized as follows: 0–50 min, 10–30% B. The DAD was used in this study to conduct spectral scans over the range of 200–400 nm. The detection wavelengths were set to 300 nm to determine and quantify multiple bioactive components. The developed HPLC method was validated via linearity, intra- and inter-day precision and accuracy, the limit of detection (LOD), and the limit of quantification (LOQ) according to the International Council for Harmonization (ICH) guidelines and the Korean Ministry of Food and Drug Safety (MFDS) guidelines [25].

### 2.8. Determination of Total Phenolic Content (TPC) and Total Flavonoid Content (TFC)

The TPC content in the *A. quinata* leaves was quantified using Folin–Ciocalteu’s method with minor modifications [26]. Briefly, 25 μL of the extract solution or gallic acid standard solution with different concentrations (0–200 μg/mL), Folin–Ciocalteu’s reagent (1 to 3 diluted with water, 25 μL), and 125 μL water were added to a 96-well plate (Costar, Corning, NY, USA), then the mixture was incubated for 8 min in the dark at room temperature. After that, 10% (*w/w*) sodium carbonate (25 μL) was added to the mixture and incubated for 60 min. The absorbance was measured at 765 nm using a Tecan infinite^®^ F200 microplate reader (Tecan Group, Ltd., Männedorf, Switzerland), and the results were presented as mg GAE (gallic acid equivalents)/g DW (dry weight). The total flavonoid content (TFC) was determined using the method reported by Subbiah et al. [27]. TFC was expressed as mg quercetin (quercetin equivalents)/g DW.

### 2.9. Statistical Analysis

Hierarchical cluster analysis (HCA) combined with heatmap analysis, PCA, and PLS-DA were performed using MetaboAnalyst 5.0 to perform multivariate analysis to clarify the samples from the different locations [28]. One-way analysis of variance (ANOVA) and *t*-test were applied to identify the statistically significant data via GraphPad Prism 8.0.2 (GraphPad Software, Inc., LaJolla, CA, USA).

## 3. Results and Discussion

### 3.1. Cell Viability Study (MTT Assays)

To determine whether the *A. quinata* leaves ethanol extracts have potential inhibition effects on the viability of HUVECs, the HUVECs were treated with different concentrations (1, 2.5, 5, 10 μg/mL) of the extract for 24 h, and cell viability was evaluated. The cell viability of the *A. quinata* leaves extracts at the test concentrations was higher than 90%, indicating no toxicity against HUVECs at these concentrations (Figure S2). 

### 3.2. A. quinata Leaves Suppress the Expression Levels of Adhesion Molecules in LPS-Stimulated HUVECs

**Figure 1:**
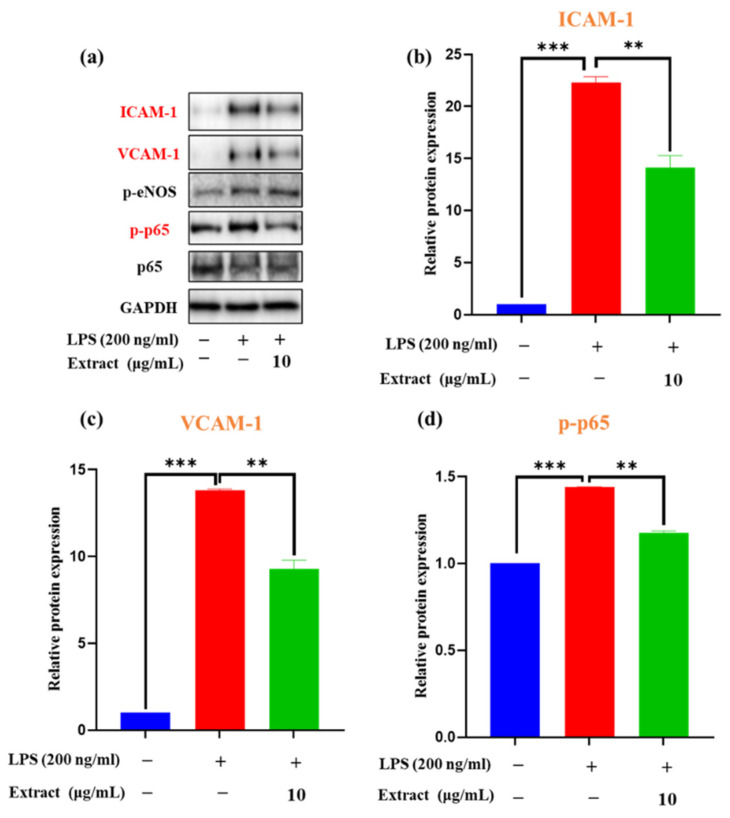
Effects of *A. quinata* extract on LPS-induced HUVEC activation and expression of (**a**) adhesion molecules and Nuclear factor-kappa B (NF-κB) signaling pathway-related factors of p-p65; the relative protein expression of (**b**) ICAM-1, (**c**) VCAM-1, and (**d**) p-p65. The experiment was repeated three times and similar results were acquired. Data are expressed as the means ± SDs. ** *p* < 0.01 vs. the LPS-treated group, *** *p* < 0.001 vs. the control cell group.

The effects of *A. quinata leaves* on the expression of VCAM-1 and ICAM-1 were evaluated by western blotting in LPS-stimulated HUVECs, which are momentous adhesion molecules that play significant roles in the initial adhesion and subsequent trans-endothelia migration of leukocytes into inflamed vessels. The expression of both ICAM-1 and VCAM-1 by LPS induction of HUVECs was substantially increased compared with the control treatment (Figure 1a–c). Interestingly, pretreated LPS-treated HUVECs with *A. quinata* leaves significantly suppressed the expression of VCAM-1 and ICAM-1 at the concentration of 10 μg/mL (Figure 1b,c). A previous study demonstrated that fresh spinach juice could downregulate the expression levels of ICAM-1 and VCAM-1, revealing its potential as a valuable therapeutic agent for the improvement of cardiovascular function [19]. Therefore, *A. quinata* leaves could be used as a natural resource for the treatment of cardiovascular disease.

### 3.3. Effects of A. quinata Leaves on LPS-Stimulated NF-κB Activation

Chronic inflammation has been found to be linked to oxidative stress [29]. Superoxide anion and other reactive oxygen species are able to stimulate NF-κB, a transcription factor that is associated with various inflammatory disorders [30]. NF-κB activates a variety of pro-inflammatory cytokines such as tumor necrosis factor-alpha and interleukin-1 [31,32]. Phosphorylation of the NF-κB p65 subunit, particularly in Ser536, is implicated in the increment of NF-κB activity and plays an essential role in the transcription of regulatory adhesion molecules in response to inflammatory stimuli [33]. Moreover, LPS stimulation can cause the activation of several important intracellular signaling molecules, which have been to activate the NF-κB pathway and mitogen-activated protein kinase in endothelial cells [31]. Thus, we evaluated the effects of *A. quinata* leaves on LPS-induced NF-κB activation. Western blot analysis results revealed that LPS could significantly induce NF-κB p65 phosphorylation. Interestingly, *A. quinata* leaves extracts at the concentration of 10 μg/mL inhibited p65 phosphorylation and further decreased protein activation (Figure 1d). Overall, all the above results prove that *A. quinata* leaves are a promising raw material to produce functional food to improve cardiovascular function. 

### 3.4. Optimization of HPLC Analytical Conditions and Extraction Parameters

Moreover, the phytochemical profiling of *A. quinata* leaves, as well as the quantification of their bioactive components and the establishment of a suitable HPLC method to evaluate leaf quality, were subsequently performed. Mobile phase compositions under chromatographic conditions were optimized to obtain the desired resolution and active components in *A. quinata* leaves. Several compositions of mobile phases were evaluated, including acetonitrile-aqueous, methanol-aqueous, 0.1% formic acid acetonitrile—0.1% formic acid aqueous, and 0.1% acetic acid acetonitrile—0.1% acetic acid aqueous. The composition of 0.1% formic acid acetonitrile—0.1% formic acid aqueous showed the best results in regards to resolution and the shape of the adjacent peaks. Different gradient elution programs, detection wavelengths (210, 254, 280, 300 and 360 nm), flow rates (0.8, 1.0 and 1.2 mL/min), and column temperatures (25 °C, 30 °C, and 35 °C) were optimized. The final optimized chromatographic parameters were: detection wavelength of 300 nm; flow rate of 1 mL/min; column temperature of 30 °C; and 50 min for each run (Figure S3).

Ultrasonic-assisted extraction and 70% food-grade alcohol were selected as the extraction method and extraction solvent, respectively, due to their environment-friendly and human-friendly properties. Chlorogenic acid and its derivative have been proved to prevent cardiovascular disease by increasing high-density lipoprotein [34]. Isochlorogenic acid has strong anti-atherosclerotic activity, which may protect endothelial cells and suppress vascular smooth muscle cell proliferation and migration [35]. Flavonoids have excellent protective abilities against cardiovascular disease, cancer, obesity, neurodegenerative diseases, diabetes, and several other diseases, especially rutin, which has been documented to offer potential cardioprotective effects in cardiac remodeling [36,37]. In addition, the leaves of *A. quinata* have been widely reported to be rich in phenolic and flavonoid compounds [7]. Therefore, neochlorogenic acid, chlorogenic acid, cryptochlorogenic acid, rutin, and isochlorogenic acid C were selected as the potential marker compounds for the evaluation of the quality of *A. quinata* leaves. The effect of the solvent-to-material ratio on the yields of the marker compounds was investigated. The result showed the maximum extraction yield of the marker compounds to be a 70% ethanol/sample ratio of 80:1 mL/g (Figure S4a). It was also observed that a slight decrease in the yield of the marker compounds occurred after the solvent-to-material ratio went above 80:1 mL/g. When the solvent-to-material ratio increased beyond a certain amount, the yield of the bioactive compounds decreased due to the attenuation of the ultrasonic waves; that is, the active portion was restricted to a zone located near the ultrasound probe, while other dissolved compounds increased in the leaf samples, thus hindering the dissolution of the marker compounds [38,39]. At the same time, the prolonged exposure of the extracted phenolic and flavonoid compounds to sonication might have caused degradation and structural destruction due to the generation of free radicals by the ultrasound waves and other components, which were extracted, then reacted or polymerized with the target compounds [38,40,41]. The effect of ultrasonic time on the bioactive component yield was also evaluated from 0–120 min. The content of the marker compounds increased as ultrasonication time increased from 0–80 min but decreased after 80 min, which may have been caused by chemical structure degradation induced by prolonged ultrasonic treatment [42,43]. Given these results, a period of 80 min was selected for the ultrasonic treatment (Figure S4b).

### 3.5. Method Validation and Quantitative Analysis

The developed method was validated in terms of linearity, LOD, LOQ, precision, accuracy, repeatability, and recovery. The linearity was evaluated by building calibration curves with peak areas of the target compounds at different concentrations under optimized HPLC–DAD conditions. The LOD and LOQ were evaluated based on the signal-to-noise ratios of 3:1 and 10:1, respectively. The results of LOD and LOQ ranged from 0.16–0.62 μg/mL, and 0.48–1.99 μg/mL (Table 1), respectively, which showed excellent sensitivity. Precision and accuracy were determined via five replicate determinations of three different concentrations (low, middle, and high) of the marker compound standards. These data were articulated as the relative standard deviation (RSD). The results demonstrated that the intra-day and inter-day precision for the investigated compounds were 0.2–2.3% and 0.6–2.8%, respectively. The accuracy of the optimized method ranged from 97.1–107.2% (Table 2). The recovery was evaluated using the standard addition method, and the results of all compounds were acceptable (Table 2). Repeatability was employed to investigate the stability of the HPLC–DAD method after consecutive injections. The RSD of the retention time and contents of the marker compounds ranged from 0.7–2.8% and 0.00–0.04%, respectively, which were within the acceptable range of the guideline [34]. 

### 3.6. Quantitative Analysis of Marker Compounds, TPC, and TFC in A. quinata Leaves from Different Cities

**Figure 2:**
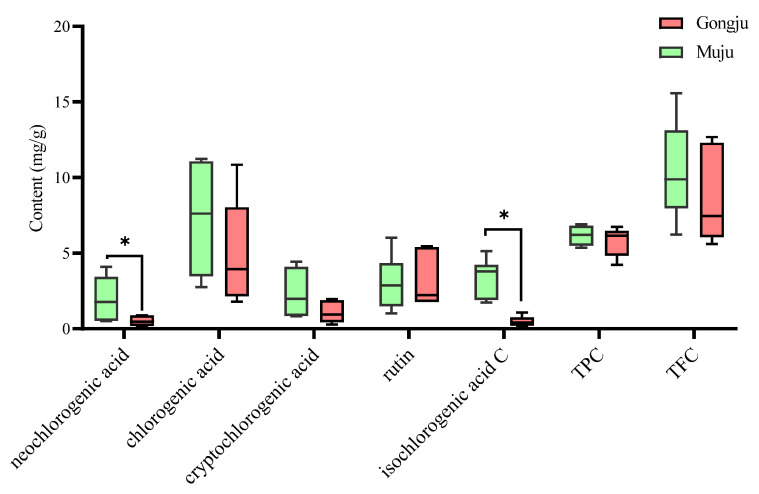
Boxplot analysis of marker compounds in 14 batches sample from Gongju and Muju. TPC: total phenolic content; TFC: total flavonoid content. * *p* < 0.05.

The HPLC–DAD method was subsequently used for the comprehensive quality assessment of 14 *A. quinata* leaves samples. The results of the quantitative determination are shown in Figure 2, which suggests that the contents of the chlorogenic acid, cryptochlorogenic acid, and rutin were not significantly different between the cities of Muju and Gongju (*p* > 0.05). Additionally, TFC and TPC did not show any significant differences between the two cities (*p* > 0.05). In contrast, there were remarkable differences in the amounts of neochlorogenic acid and isochlorogenic acid C between the two cities (*p* < 0.05). Interestingly, isochlorogenic acid C is considered the active compound in *A. quinata* stems that inhibits the differentiation of 3T3-L1 cells, thus enhancing the activation of adenosine monophosphate-activated protein kinase phosphorylation for the treatment of obesity and hyperlipemia [17]. Moreover, phenolic acids have been known to prevent pathological processes, such as diabetes, cardiovascular diseases, obesity, and atherogenic processes [44,45,46,47]. Thus, *A. quinata* leaves could be a potential agent in the development of functional foods that can treat obesity and cardiovascular disease. Since we have shown that the number of bioactive compounds can differ according to location, the cultivation regions should be considered to ensure the safety and validity of *A. quinata* leaves [34]. 

### 3.7. Establishment of the HPLC Fingerprints of A. quinata Leaves

**Figure 3:**
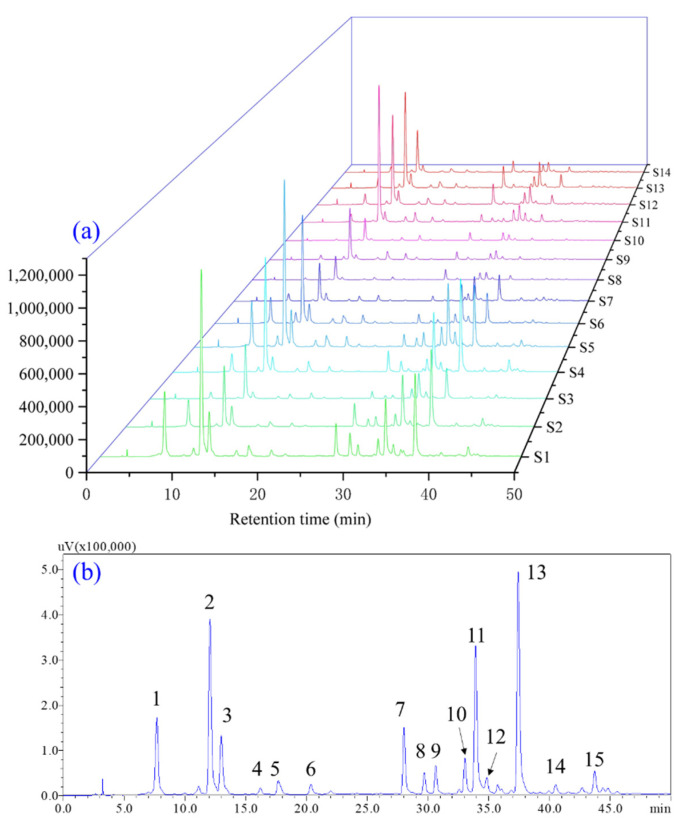
The typical (**a**) HPLC fingerprints of 14 *A. quinata* leaves samples from different cities and reference fingerprint of *A. quinata* leaves (**b**). 1: neochlorogenic acid; 2: chlorogenic acid; 3: cryptochlorogenic acid; 4: unknown; 5: 5-*O*-*p*-coumaroylquinic acid; 6: 5-*O*-feruloylquinic acid; 7: rutin; 8: quercetin-3-*O*-glucoside; 9: unknown; 10: nictotiflorin, 11: isochlorogenic acid A, 12: astragalin; 13: isochlorogenic acid C, 14: unknown; 15: unknown.

Under the optimal HPLC–DAD conditions, the typical HPLC fingerprints of the 14 samples had similar HPLC profiles (Figure 3a). Based on the fingerprints of *A. quinata* leaves from the different cities, the S2 chromatogram was selected as a representative chromatogram since it contains the most investigated ingredients (Figure 3b). When considering the peak responses, separation, and retention times, 15 peaks responsible for more than 90% of the total peak area were selected for further analysis. Among these, 11 peaks were assigned as neochlorogenic acid (peak 1), chlorogenic acid (peak 2), cryptochlorogenic acid (peak 3), 5-*O*-*p*-coumaroylquinic acid (peak 5), 5-*O*-feruloylquinic acid (peak 6), rutin (peak 7), quercetin-3-*O*-glucoside (peak 8), nictotiflorin (peak 10), isochlorogenic acid A (peak 11), astragalin (peak 12), and isochlorogenic acid C (peak 13) by comparing their retention time, MS spectrum (in the positive and negative modes), fragmentation ions, and UV spectrum with those of standard compounds and available references [7,48,49]. The other four peaks remained unknown.

### 3.8. HCA Multi-Fingerprints of A. quinata Leaves

**Figure 4:**
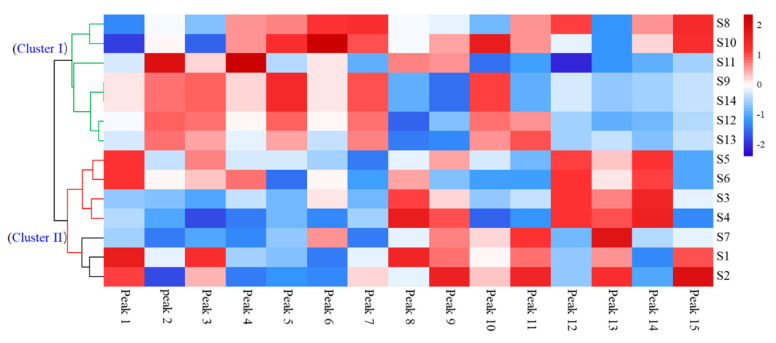
Dendrogram and heatmap of *A. quinata* leaves from Gongju and Muju.

HCA is a clustering technology that analyzes the similarity and differences among different groups via an algorithm to obtain a dendrogram, which can directly show the differences between samples through color changes on a heatmap [50,51]. Thus, a heatmap cluster analysis was performed to evaluate the similarity and difference of the bioactive component profiles in the *A. quinata* leaves (Figure 4). The results showed that the 14 samples could be classified into two clusters: samples 8–14 from Gongju were gathered in Cluster I with the lower contents of peak 1 (neochlorogenic acid), peak 3 (cryptochlorogenic acid), and peak 13 (isochlorogenic acid C); those from Muju were distributed in Cluster II and exhibited a higher content of peak 9 (unknown), peak 12 (astragalin), and peak 13 (isochlorogenic acid C). The different contents of these bioactive components may be a result of the different growth conditions in their respective environments [52,53].

### 3.9. PCA and PLS-DA Analysis of Multi-Fingerprints

To further examine the variation in the common components of the *A. quinata* leaves, PCA and PLS-DA were applied to discriminate the group via their fingerprints and to find potential markers. The performance of the PLS-DA models was evaluated by 10-fold cross-validation. The loading scatter plot and variable importance in projection (VIP) scores were used to select the potential markers.

**Figure 5:**
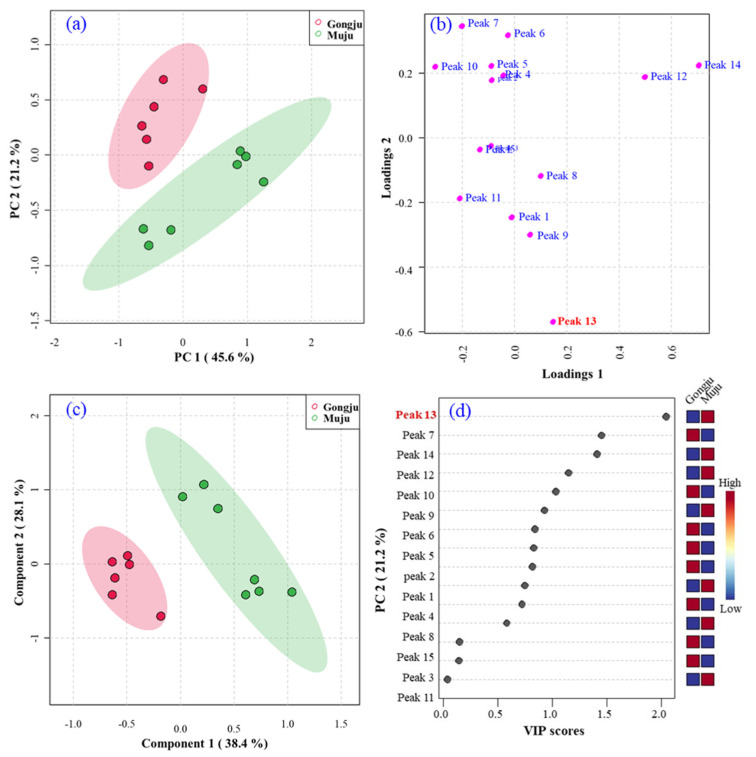
(**a**) Unsupervised PCA score plot, (**b**) loading plot, (**c**) supervised PLS-DA score plot and (**d**) VIP score plot based on the HPLC fingerprints of *A. quinata* leaves from Gongju and Muju.

The two major principal components (PC1 and PC2), accounting for 66.8% of the total variance, were selected to produce the PCA score plot of common components in all samples. As shown in Figure 5a, the PCA score plot separated the samples into two clusters (Cluster I: Gongju; Cluster II: Muju), which was in accordance with the heatmap cluster analysis results. The loading scatter plot (Figure 5b) showed that peak 13 was located far from the y-axis, suggesting that this component could be the marker compound among the bioactive components in the *A. quinata* leaves. PLS-DA was performed to investigate the source of the differences between the two clusters. The R2 and Q2 values of the cross-validation were 0.99 and 0.87, which demonstrates that the PLS-DA model was stable and valid and can be used to discriminate the samples (Figure 5c) [54]. The VIP score (Figure 5d) also illustrated that peak 13 (isochlorogenic acid C) was the most contributing marker compound for the discrimination of *A. quinata* leaves from Gongju and Muju (VIP > 1.5).

## 4. Conclusions

Taken together, in the current study, we proved that *A. quinata* leaves significantly inhibited LPS-stimulated adhesion molecules VCAM-1 and ICAM-1 expression. In addition, an HPLC–DAD analytical method was established for the comprehensive quality assessment of *A. quinata* leaves. The developed method was applied for fingerprint profiling of the leaves and quantitative analysis of five representative components. HPLC–DAD–ESI–MS/MS was further applied to tentatively identify prominent peaks in the samples. The results demonstrated that the *A. quinata* leaves from the two regions shared a similar HPLC pattern; all contained 15 characteristic peaks with high concentrations of chlorogenic acid derivatives, although they differed in amounts. According to the establishment of the fingerprints, the 14 samples were successfully clustered or discriminated using chemometric tools (HCA heatmap, PCA, and PLS-DA). Our study demonstrated that the developed analytical method was reliable and could be used as a simple tool for the comprehensive quality control of *A. quinata* leaves. 

## Figures and Tables

**Table 1:** Regression equation, linearity, LODs and LOQs of the investigated components.

Compounds	Linearity Range (μg/mL)	R ^a^	Equation ^b^	LOD (μg/mL)	LOQ (μg/mL)
Neochlorogenic acid	5–200	1.0000	y = 29.1x − 9.9	0.17	0.50
Chlorogenic acid	50–800	0.9997	y = 26.0x − 67.8	0.16	0.48
Cryptohlorogenic acid	5–200	0.9999	y = 19.8x − 5.5	0.17	0.49
Rutin	25–600	0.9998	y = 8.4x + 1.2	0.39	1.28
Isochlorogenic acid C	5–200	0.9999	y = 33.3x − 3.7	0.62	1.99

^a^ y and x are the peak area (in thousands) and concentrations of the analytes. ^b^ R = correlation coefficient, *n* = 6.

**Table 2:** Precision, accuracy, recovery and repeatability of the investigated components.

Compounds	Precision		Accuracy		Recovery ^a^ (%)	Repeatability	
	Intra-Day (%RSD)	Inter-Day (%RSD)	Intra-Day (%)	Inter-Day (%)		Retention Time (%RSD)	Content (%RSD)
Neochlorogenic acid	0.4–0.6	1.6–1.9	97.1–98.6	97.5–98.4	97.5–99.8	2.8	0.04
Chlorogenic acid	0.3–2.2	1.7–2.4	98.6–104.3	98.9–105.2	98.3–99.6	1.6	0.02
Cryptohlorogenic acid	0.2–0.8	1.2–2.8	98.9–100.9	99.8–107.2	97.9–103.3	2.5	0.02
Rutin	0.4–2.3	0.9–2.1	100.2–100.4	98.2–103.1	97.0–98.7	0.7	0.00
Isochlorogenic acid C	0.9–1.3	0.6–2.4	98.2–103.9	97.9–106.2	99.8–104.1	1.4	0.10

^a^ Recovery (%) = (Found concentration − original concentration)/Spiked concentration × 100.

## Data Availability

Data are contained within the article and Supplementary Materials.

## Supplementary Materials

The following supporting information can be downloaded at: https://www.mdpi.com/article/10.3390/molecules27144636/s1, Figure S1: The picture of *A. quinata* leaves and stems. Note: the leaves were separated from stems and used for preparation of *A. quinata* leaves extracts; Figure S2: Effects of *A. quinata* leaves extract on HUVECs viability; Figure S3: The typical HPLC-UV chromatograms of (**a**) mixture of standards and (**b**) sample solution measured at 300 nm. (1: neochlorogenic acid; 2: chlorogenic acid; 3: cryptochlorogenic acid; 4: rutin; 5: isochlorogenic acid C); Figure S4: Effect of (**a**) solvent to material (10:1–50:1 mL/g) and (**b**) sonicated treatment time (0–120 min) on the yield of the total content of marker components in the single-factor test; Table S1: Source of 14 batches *A. quinata* leaves and stems. Note: the leaves were separated from stems and used for preparation of *A. quinata* leaves extracts.

## Sample Availability

Samples of the compounds are not available from the authors.
