# Supplementary material for: Phenolic Profile and Fingerprint Analysis of Akebia quinata Leaves Extract with Endothelial Protective Activity
Source: Molecules. 2022 Jul 20;27(14):4636. doi: 10.3390/molecules27144636 (PMC9316754; doi:10.3390/molecules27144636)
Supplement: Supplementary file 1 [file molecules-27-04636-s001.zip › molecules-1798582-supplementary.pdf]

molecules

*Supplementary Materials*

# Phenolic Profile and Fingerprint Analysis of *Akebia quinata* Leaves Extract with Endothelial Protective Activity

**Dan Gao [1], Chong-Woon Cho [2], Jin-Hyeok Kim [2], Haiying Bao [3], Hyung-Min Kim [2], Xiwen Li [1,*] and Jong-Seong Kang [2,*]**

[1] Institute of Chinese Materia Medica, China Academy of Chinese Medical Sciences, Beijing 100700, China; gaodan521361@hotmail.com
[2] College of Pharmacy, Chungnam National University, Daejeon 34134, Korea; chongw113@naver.com (C.-W.C.); oojh52@cnu.ac.kr (J.-H.K.); kimhm@cnu.ac.kr (H.-M.K.)
[3] College of Chinese Medicinal Materials, Jilin Agriculture University, Changchun 130118, China; baohaiying2008@126.com
* Correspondence: xwli@icmm.ac.cn (X.L.); kangjss@cnu.ac.kr (J.-S.K.); Tel.: +86-10-8408-4107 (X.L.); +82-42-821-5928 (J.-S.K.)

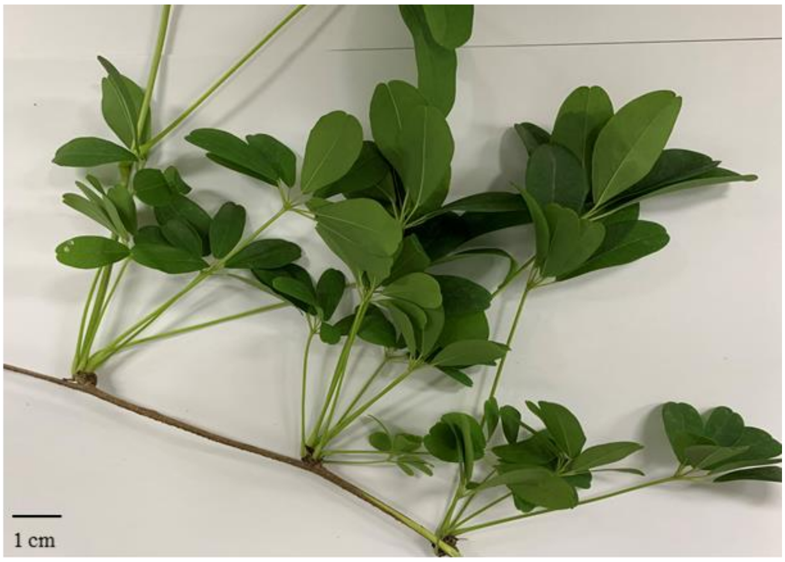


**Figure S1.** The picture of *A. quinata* leaves and stems. Note: the leaves were separated from stems and used for preparation of *A. quinata* leaves extracts.

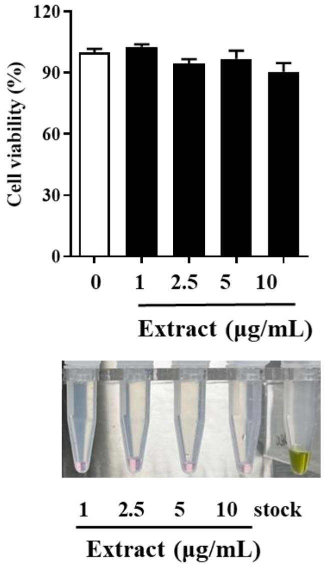


**Figure S2.** Effects of *A.quinata* leaves extract on HUVECs viability.

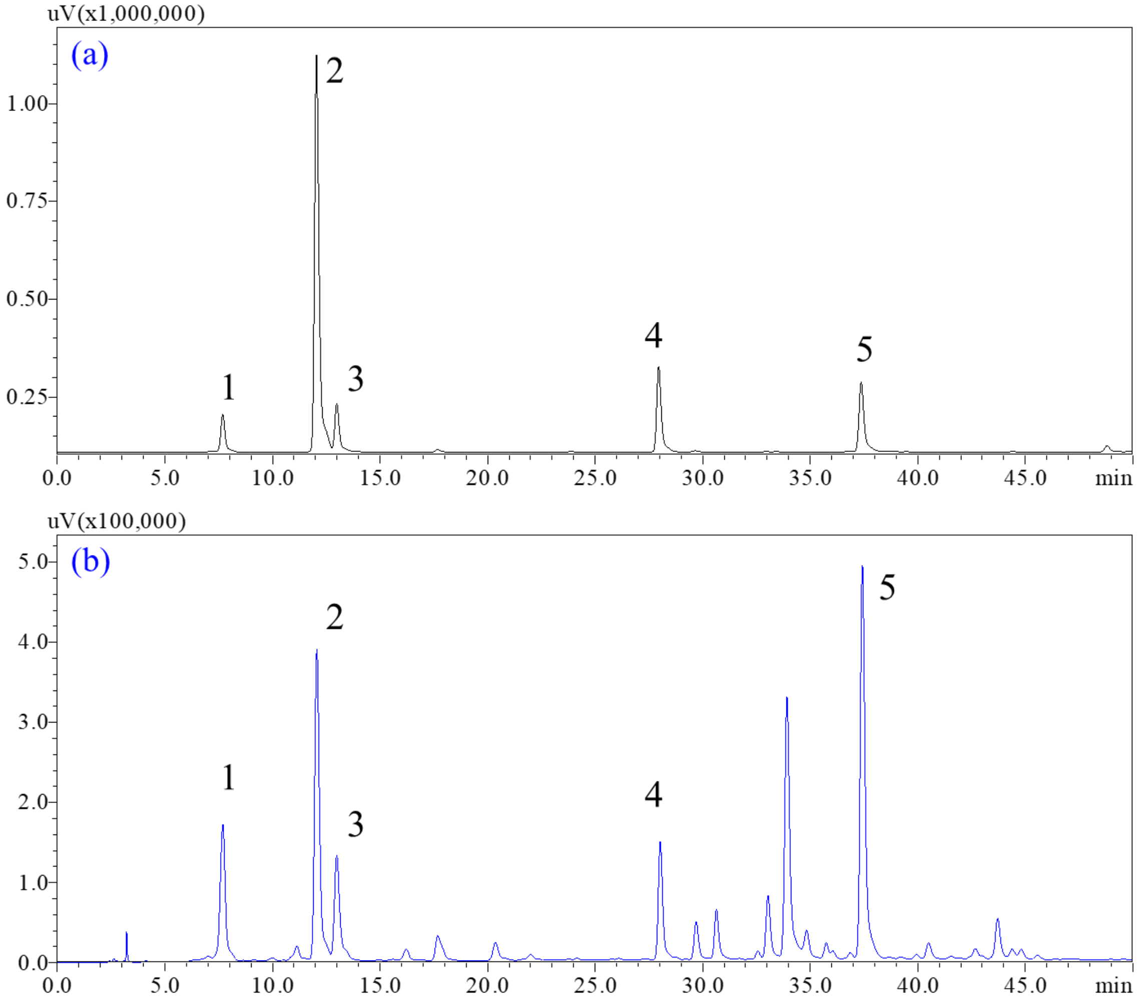


**Figure S3.** The typical HPLC-UV chromatograms of (**a**) mixture of standards and (**b**) sample soluTable 300. nm. (1: neochlorogenic acid; 2: chlorogenic acid; 3: cryptochlorogenic acid; 4: rutin; 5: isochlorogenic acid C).

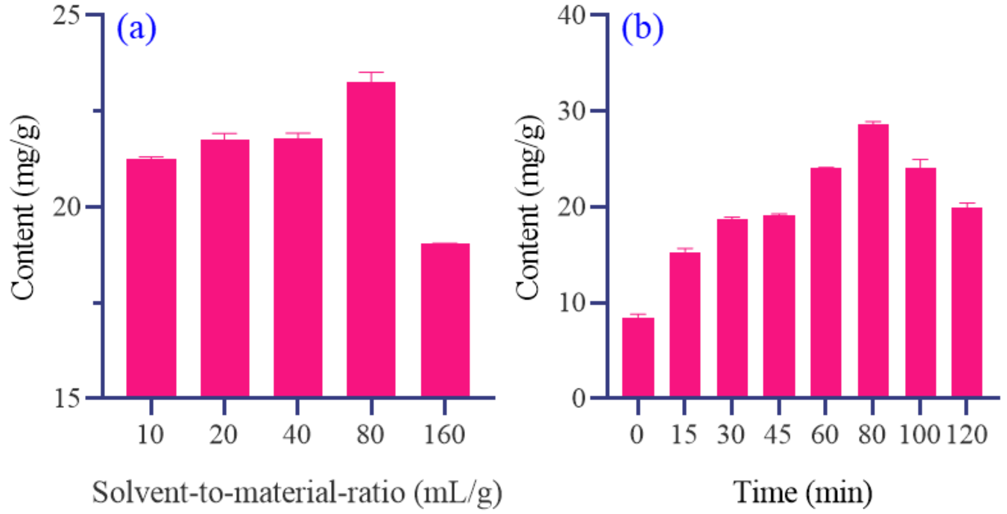


**Figure S4.** Effect of (**a**) solvent to material (10:1–50:1 mL/g) and (**b**) sonicated treatment time (0–120 min) on the yield of the total content of marker components in the single-factor test.

**Table S1.** Source of 14 batches *A. quinata* leaves and stems. Note: the leaves were separated from stems and used for preparation of *A. quinata* leaves extracts.

| Code | Source | Collect Time |
|---|---|---|
| S1 | Muju, Korea | 2021.08.09 |
| S2 | Muju, Korea | 2021.08.09 |
| S3 | Muju, Korea | 2021.08.09 |
| S4 | Muju, Korea | 2021.08.09 |
| S5 | Muju, Korea | 2021.08.09 |
| S6 | Muju, Korea | 2021.08.09 |
| S7 | Muju, Korea | 2021.08.09 |
| S8 | Gongju, Korea | 2021.07.30 |
| S9 | Gongju, Korea | 2021.07.30 |
| S10 | Gongju, Korea | 2021.07.30 |
| S11 | Gongju, Korea | 2021.08.03 |
| S12 | Gongju, Korea | 2021.09.03 |
| S13 | Gongju, Korea | 2021.09.03 |
| S14 | Gongju, Korea | 2021.09.03 |
